# Genome editing in ubiquitous freshwater Actinobacteria

**DOI:** 10.1128/aem.00865-24

**Published:** 2024-10-16

**Authors:** Nachiketa Bairagi, Jessica L. Keffer, Jordan C. Heydt, Julia A. Maresca

**Affiliations:** 1 Department of Civil and Environmental Engineering, University of Delaware, Newark, Delaware, USA; 2 Department of Earth Sciences, University of Delaware, Newark, Delaware, USA; 3 School of Marine Science and Policy, University of Delaware, Newark, Delaware, USA; Washington University in St. Louis, St. Louis, Missouri, USA

**Keywords:** genome editing, transformation, selectable markers, freshwater, Actinobacteria, carotenoids

## Abstract

Development of genome-editing tools in diverse microbial species is an important step both in understanding the roles of those microbes in different environments, and in engineering microbes for a variety of applications. Freshwater-specific clades of Actinobacteria are ubiquitous and abundant in surface freshwaters worldwide. Here, we show that *Rhodoluna lacicola* and *Aurantimicrobium photophilum*, which represent widespread clades of freshwater Actinobacteria, are naturally transformable. We also show that gene inactivation via double homologous recombination and replacement of the target gene with antibiotic selection markers can be used in both strains, making them convenient and broadly accessible model organisms for freshwater systems. We further show that in both strains, the predicted phytoene synthase is the only phytoene synthase, and its inactivation prevents the synthesis of all pigments. The tools developed here enable targeted modification of the genomes of some of the most abundant microbes in freshwater communities. These genome-editing tools will enable hypothesis testing about the genetics and (eco)physiology of freshwater Actinobacteria and broaden the available model systems for engineering freshwater microbial communities.

**IMPORTANCE:**

To advance bioproduction or bioremediation in large, unsupervised environmental systems such as ponds, wastewater lagoons, or groundwater systems, it will be necessary to develop diverse genetically amenable microbial model organisms. Although we already genetically modify a few key species, tools for engineering more microbial taxa, with different natural phenotypes, will enable us to genetically engineer multispecies consortia or even complex communities. Developing genetic tools for modifying freshwater bacteria is particularly important, as wastewater, production ponds or raceways, and contaminated surface water are all freshwater systems where microbial communities are already deployed to do work, and the outputs could potentially be enhanced by genetic modifications. Here, we demonstrate that common tools for genome editing can be used to inactivate specific genes in two representatives of a very widespread, environmentally relevant group of Actinobacteria. These Actinobacteria are found in almost all tested surface freshwater environments, where they co-occur with primary producers, and genome-editing tools in these species are thus a step on the way to engineering microbial consortia in freshwater environments.

## INTRODUCTION

Diversification of genetically amenable bacterial systems is critically important for advancing bioproduction, bioprospecting, and biodegradation. The workhorse model organisms such as *Escherichia coli* or *Saccharomyces cerevisiae* are relatively easily modified, but starting with an organism that already has all or some of the desired metabolic capabilities or environmental tolerances would mean that fewer genomic modifications are required (1–3). To have a library of genetically tractable organisms that can operate in the full range of environmental conditions, we need a broad phylogenetic and phenotypic range of microbes that can be genetically engineered.

In freshwater environments, freshwater-specific clades of low-GC Actinobacteria comprise up to 60% of the bacteria in surface waters and likely mediate much of the heterotrophic conversion of dissolved organic carbon to CO_2_ and buried biomass (4–16). Globally, metagenomic analyses in combination with geochemical measurements have indicated that freshwater Microbacteriaceae play key roles in carbon, nitrogen, phosphorus, and sulfur cycling in lakes and ponds, from coastal lagoons in South America to bogs and ponds in the American Midwest, to alpine lakes in Europe and Asia (7, 8, 16, 17). Genome analysis of these freshwater strains indicates that they form coherent clades within the Microbacteriaceae family of Actinobacteria and have quite similar, very small, genomes (<2 Mbp) (18–24). Despite their limited genomic resources, they are keystone species in freshwater metabolic networks (18, 25–29), and may also be useful bioindicators of trophic status (30).

Because these clades are ubiquitous and abundant, understanding their genetics, metabolism, and ecophysiology is fundamental to understanding freshwater biogeochemistry. However, no genome-editing tools yet exist for any of the freshwater-specific clades—the closest relatives in which genes can be inactivated or heterologously expressed are phytopathogenic *Clavibacter* spp. (31–33), which are high-GC species with larger genomes (34). Investigation into the functions of specific genes in freshwater Actinobacterial clades has thus relied on heterologous expression in model organisms such as *E. coli* until now (35, 36).

A broad understanding of how and why freshwater Actinobacteria dominate in diverse freshwater environments will require systems-level tools for genome editing and analysis. Therefore, our goal was to develop tools for targeted gene inactivation in *Rhodoluna (R.) lacicola* strain MWH-Ta8, representative of the Luna-1 clade of freshwater Actinobacteria (12, 19), and *Aurantimicrobium* (*A*.) *photophilum* strain MWH-Mo1, representative of the Luna2/acIII clade (20). Isolates from these clades tend to be brightly colored due to carotenoid pigments (17). The *crtB* gene, encoding the first committed step in carotenoid biosynthesis, was chosen for these proof-of-principle experiments because of the ubiquity of carotenoids in these strains (17, 35), the lack of existing biochemical data about pigment biosynthesis in freshwater Actinobacteria (37) and because we previously isolated a spontaneous mutant in the *crtB* gene of *R. lacicola*. Inactivation of this gene via a point mutation that introduces an early stop codon caused a loss of pigmentation but had no effect on growth (38). We show that *R. lacicola* can take up DNA via natural transformation with either linear and plasmid DNA or via electroporation with plasmid DNA and that *A. photophilum* can take up linear DNA via natural transformation. Targeted gene inactivation is possible in both strains using antibiotic selection for double homologous recombination, enabling the characterization of the roles of specific genes and pathways in the ecophysiology of this group of keystone organisms in freshwater environments.

## MATERIALS AND METHODS

### Strains and growth conditions

Genome-editing tools were developed for two cultures: *Rhodoluna lacicola* MWH-Ta8 (19) and *Aurantimicrobium photophilum* MWH-Mo1 (20). The *R. lacicola* culture is available at the DSMZ culture collection under strain no. 23834; its genome sequence is available at NCBI under accession no. GCA_000699505.1. The *A. photophilum* culture is available at the DSMZ under strain no. DSM 107758 and its genome sequence is available at NCBI under accession no. GCA_003194085.1. Both strains were grown aerobically either in NSY medium [per liter: 10 mL 100× inorganic basal medium with 1 g L^−1^ each nutrient broth, soytone, and yeast extract (12)] supplemented with vitamin B12 (1 mg mL^−1^) and sodium thiosulfate (50 mM), or in minimal media composed of, per liter, 10 mL 100× inorganic basal medium (12) with 1 g NaCl, 0.4 g MgCl_2_ × 6H_2_O, 0.1 g CaCl_2_ × 2H_2_O, 0.2 g KH_2_PO_4_, 0.5 g KCl, 1 g NH_4_Cl, and 50 µM thiosulfate. The pH was adjusted to 8.0, and after autoclaving, the medium was supplemented with (per liter) 40 mg L-asparagine, 40 mg L-cysteine, 1 mL 1,000 × 8-vitamin mix, 1 µg L^−1^ vitamin B12, and 0.1% (vol/vol) D-fructose. The 8-vitamin mix was composed of 100 mg mL^−1^ each thiamine-HCl, D-Calcium pantothenate, folic acid, nicotinic acid, 4-aminobenzoic acid, pyridoxine-HCl, lipoic acid, and biotin.

Solid NSY and minimal medium had the same compositions, solidified with 15 g L^−1^ agar. For *R. lacicola*, the medium was supplemented with ampicillin (20 µg mL^−1^), chloramphenicol (20 µg mL^−1^), kanamycin (30 µg mL^−1^), or tetracycline (10 µg mL^−1^), as appropriate. For *A. photophilum*, the medium was supplemented with ampicillin (75 µg mL^−1^). Cells in liquid culture were grown at 30°C with shaking (~150 rpm); cells on solid medium were incubated at room temperature (~26°C). When applicable, growth in liquid culture was quantified by measuring optical density (OD) at 600 nm using a Fisher Scientific BioMate 3S UV-Vis spectrophotometer.

For plasmid propagation, *E. coli* strain NEB 5-alpha (New England Biolabs, catalog # C2987H) was grown on liquid or solid LB medium supplemented with ampicillin (100 µg mL^−1^), chloramphenicol (34 µg mL^−1^), kanamycin (30 µg mL^−1^), or tetracycline (15 µg mL^−1^), as appropriate.

### Antibiotic sensitivity assays

*R. lacicola* was grown in liquid NSY medium until late exponential phase, then harvested by centrifugation (5,000 rpm, 20 min) and resuspended in NSY medium. Cell suspensions were spread on solid NSY medium and sensitivity to the antibiotics ampicillin, chloramphenicol, kanamycin, streptomycin, and tetracycline was assessed by agar disk diffusion assays. Solutions of antibiotics (20 µg mL^−1^ or 200 µg mL^−1^) were applied to 6 mm paper disks (BBL item no. 231039) and air-dried. The dried disks were then placed on the *R. lacicola* lawns and the plates were incubated until growth was visible. The presence of a zone of no growth around the plates indicated sensitivity.

*A. photophilum* was grown in liquid NSY medium until late exponential phase, then harvested by centrifugation (5,000 rpm, 20 min) and resuspended in NSY medium to an optical density at 600 nm (OD_600 nm_) of 0.05. A series of 10-fold dilutions (10^−1^ to 10^−4^) were prepared. Then 1 µL of each dilution was placed on solid NSY medium with different concentrations of antibiotics and allowed to sink into the medium without spreading. All trials were done in triplicate. The plates were incubated at 29°C for 5 days, then growth was evaluated.

### Gene inactivation constructs

To inactivate *crtB* using double homologous recombination, linear constructs were synthesized using double-joint PCR (39). First, ~500 bp regions upstream and downstream of *crtB* (locus tag *rhola_00010860*) were amplified from *R. lacicola* genomic DNA using KOD-ONE polymerase master mix (Sigma Aldrich, item no. KMM-101NV) and primers Ta8_crtB_US_Fp/ Ta8_crtB_US_Rp_TcR and Ta8_crtB_DS_Fp_TcR/Ta8_crtB_DS_Rv, respectively (Table S1); the reverse primer for the upstream region and the forward primer for the downstream region also included 20–24 nucleotides homologous to the selectable marker to be used. The selectable markers were amplified using primers that were the reverse/complement of the upstream reverse and downstream forward primers from the flanking regions. A second round of PCR was then done using the products of the first three reactions as primers (39). Then, the product of the second reaction was used as the template for PCR using primers Ta8_crtB_US_Fp and Ta8_crtB_DS_Rv to amplify the full-length construct. The antibiotic resistance gene was inserted in-frame in the *crtB* locus because we have observed that *crtB* is expressed throughout the cell cycle, though expression levels vary (40).

The same approach was used to produce an inactivation construct to replace *crtB* (*AURMO_01714*) with *bla* (a beta-lactamase providing ampicillin resistance, amplified from pUC19) in *A. photophilum*. Primer sequences can be found in Table S2.

For inactivation of *cryB* (*rhola_00013030*), a knockout construct was ligated into pUC19. 500 bp regions upstream and downstream of *rhola_00013030* in the *R. lacicola* genome and the *tetR* gene from plasmid pEX18-Tc were PCR-amplified, and then joined using double-joint PCR (Table S1). This product and pUC19 were then digested with KpnI and SalI (New England Biolabs, catalog nos. R0142 and R0138, respectively). The plasmid backbone was dephosphorylated and the two fragments were ligated with T4 DNA ligase (ThermoFisher, catalog no. EL0011) and the resulting plasmid was transformed into chemically competent *E. coli* strain NEB 5-alpha (New England Biolabs, catalog #C2987H) for plasmid propagation. The resulting plasmid encoded the *tetR* gene inserted in-frame in the *rhola_00013030* locus. Plasmids were extracted and purified from a 2 mL *E. coli* culture using the GeneJet plasmid miniprep kit (Thermo Fisher, catalog #K0502).

### Transformation procedure

#### Natural transformation of *R. lacicola*

For natural transformation, *R. lacicola* cells (~10 mL) were grown in NSY on the benchtop (exposed to normal day/night light cycles) for 10 days, until the OD_600 nm_ was ~0.1, diluted to an OD_600 nm_ of ~0.01 and grown 24 h in the dark. They were then harvested by centrifugation for 10 min at 4,700 × *g* , washed two times in NSY media, then resuspended in 500 μL NSY. Linear constructs or plasmid DNA (~500 ng) were mixed with the cells and the solution was incubated without selection at room temperature overnight in the dark. The cells were transferred to solid selective NSY and incubated at 28°C until colonies were visible (~2 weeks), then individual colonies were restreaked on solid selective NSY once more. Putative mutants were screened after two rounds of colony growth on selective media.

#### Electroporation of *R. lacicola*

To test the efficacy of electroporation as a transformation method, a plasmid construct for inactivation of the gene encoding a putative CryB-type cryptochrome (*rhola_00013030*) in *R. lacicola* was made. To prepare *R. lacicola* for electroporation, cells (~50 mL) were grown in the dark in NSY amended with vitamin B12 (1 mg mL^−1^) and thiosulfate (50 mM) for 24 h. Then glycine was added to a final concentration of 1% and the cells were incubated one more hour, then harvested by centrifugation for 10 min at 4,700 × *g* and 4°C. The supernatant was discarded and the cells were washed twice in 10% ice-cold glycerol, then resuspended in 200 µL 10% glycerol, quick-frozen in a dry-ice ethanol bath, and stored at −80°C until use. Cells were thawed on ice for 10 min, then plasmid DNA (~500 ng) was mixed with cells (50 µL). The mixtures were kept on ice for 5 min and then transferred to an electroporation cuvette with a 1 mm gap. Cells were electroporated at 1.7 kV (4.8 ms) using an Eppendorf Eporator. Cells were then resuspended in 950 µL room temperature NSY media and incubated without selection at room temperature for 3 h with shaking. The cells were then transferred to solid NSY amended with tetracycline and incubated at 28°C until colonies were visible. Individual colonies were then selected and restreaked on solid selective NSY.

#### Natural transformation of *A. photophilum*

For natural transformation, *A. photophilum* cells (~100 mL) were grown in the dark in NSY for 24 h, until the OD_600 nm_ was ~0.08, then harvested by centrifugation for 10 min at 4,700 × *g,* washed two times in NSY media, and resuspended in 500 μL NSY. Linear DNA (~500 ng) was mixed with the cells and the solution was incubated without selection at room temperature for 6 h in the dark, then 6 h in the light. The cells were transferred to solid selective NSY and incubated at 28°C until colonies were visible (~10 days), then individual colonies were restreaked on solid selective NSY. Putative mutants were screened after two rounds of colony growth on selective media.

### Confirmation of insertions

For initial confirmation that double homologous recombination had occurred, replacing *crtB* with different selectable markers, individual transformant colonies were picked and resuspended in 50 uL water. 1–2 uL of this solution was used as the template for colony PCR. Primers complementary to the *R. lacicola* genome outside the upstream and downstream regions in the constructs were used to amplify the region of insertion. Amplicons from mutant strains were compared to amplicons from wild-type *R. lacicola* to confirm that the size corresponded to the expected size if *crtB* had been replaced with the correct marker gene. PCR across the region could not be used to confirm the replacement of *crtB* with *bla* in *A. photophilum* since the *crtB* and *bla* genes are very similar in size. In addition to PCR-amplification of the whole *crtB* region, PCR with one primer complementary to the region upstream of *crtB* and one complementary to the inserted beta-lactamase gene (*bla*) was used; this primer pair should generate a product of 1,475 bp in the mutant strain. Additionally, the *bla* gene was amplified from both the mutant strain and the plasmid construct.

For inactivation of the predicted CryB-type cryptochrome in *R. lacicola*, tetracycline-resistant colonies were obtained after one round of selection. However, the *tetR* cassette is similar in size to *rhola_00013030*, so PCR across the region was inconclusive. Instead, after a subsequent round of growth on non-selective medium, nested PCR using first a pair of primers complementary to the region of chromosomal DNA outside the construct, followed by amplification of *tetR* and a fragment of the wild-type gene from that product, was used to demonstrate both that *tetR* had been integrated into the chromosome at the appropriate place and that no wild-type copy of the gene remained in the cells.

### Pigment analysis

Wild-type and Δ*crtB* cultures (100 mL) of *R. lacicola* and *A. photophilum* were grown in NSY medium as described above. Cells were harvested by centrifugation at 5,000 × *g* for 15 min and resuspended in 0.4 mL high-performance liquid chromatography (HPLC)-grade acetone: methanol (7:2 vol/vol). Cells were sonicated on ice (50% duty cycle, 1 s on/off pulses) using a Fisher Scientific probe sonicator (Sonic Dismembrator model 120, probe model CL-18). The lysate was centrifuged at 12,000 × *g* for 2 min to remove cell debris, and the supernatant was filtered through a 0.2 µm polytetrafluoroethylene syringe filter (Thermo Scientific) into glass vials. Pigment extracts were then transferred to quartz cuvettes and absorption spectra from 350 to 600 nm were recorded on a Thermo Scientific BioMate 3S UV/visible spectrophotometer.

## RESULTS

### Antibiotic sensitivity of freshwater actinobacterial strains

The sensitivity of *R. lacicola* to the antibiotics ampicillin, chloramphenicol, kanamycin, streptomycin, and tetracycline was assessed by an agar disk diffusion assay. *R. lacicola* is not sensitive to streptomycin, but is sensitive to chloramphenicol, kanamycin, and tetracycline at 20 µg mL^−1^, as well as to ampicillin at 200 µg mL^−1^ (Table 1). In contrast, *A. photophilum* was resistant to chloramphenicol and kanamycin at all concentrations tested, and sensitive only to ampicillin (Table 2).

**TABLE 1:** *R. lacicola* is sensitive to chloramphenicol, kanamycin, and tetracycline^*a*^

Antibiotic	20 µg mL^−1^	200 µg mL^−1^
Chloramphenicol	S	S
Kanamycin	S	S
Streptomycin	R	R
Tetracycline	S	S

^*a*^ Sensitivity was evaluated with disk diffusion assays on solid NSY medium. *R. lacicola* was scored as resistant (“R”) if cells grew up to the edge of the disk containing the antibiotic, or sensitive (“S”) if there was a clear ring around the disk.

**TABLE 2:** *A. photophilum* is sensitive to ampicillin^*a*^

	Ampicillin				Chloramphenicol			Kanamycin		
Dilution	150	75	25	10	200	100	50	200	100	50
10^−1^	0	0	0	(+)	++	++	++	++	++	++
10^−2^	0	0	0	(+)	++	++	++	++	++	++
10^−3^	0	0	0	0	++	++	++	++	++	++
10^−4^	0	0	0	0	0	+	+	+	(+)	+

^*a*^ Sensitivity was evaluated by the growth of 1 µL culture spotted onto solid NSY medium amended with antibiotics at the indicated concentrations. Concentrations are in µg mL^−1^. “(+)” indicates very little growth; “+” indicates growth of a few colonies in the droplet area; “++” indicates confluence within the droplet area.

### Transformation and homologous recombination

#### Inactivation of *crtB* via double homologous recombination

**Fig 1:**
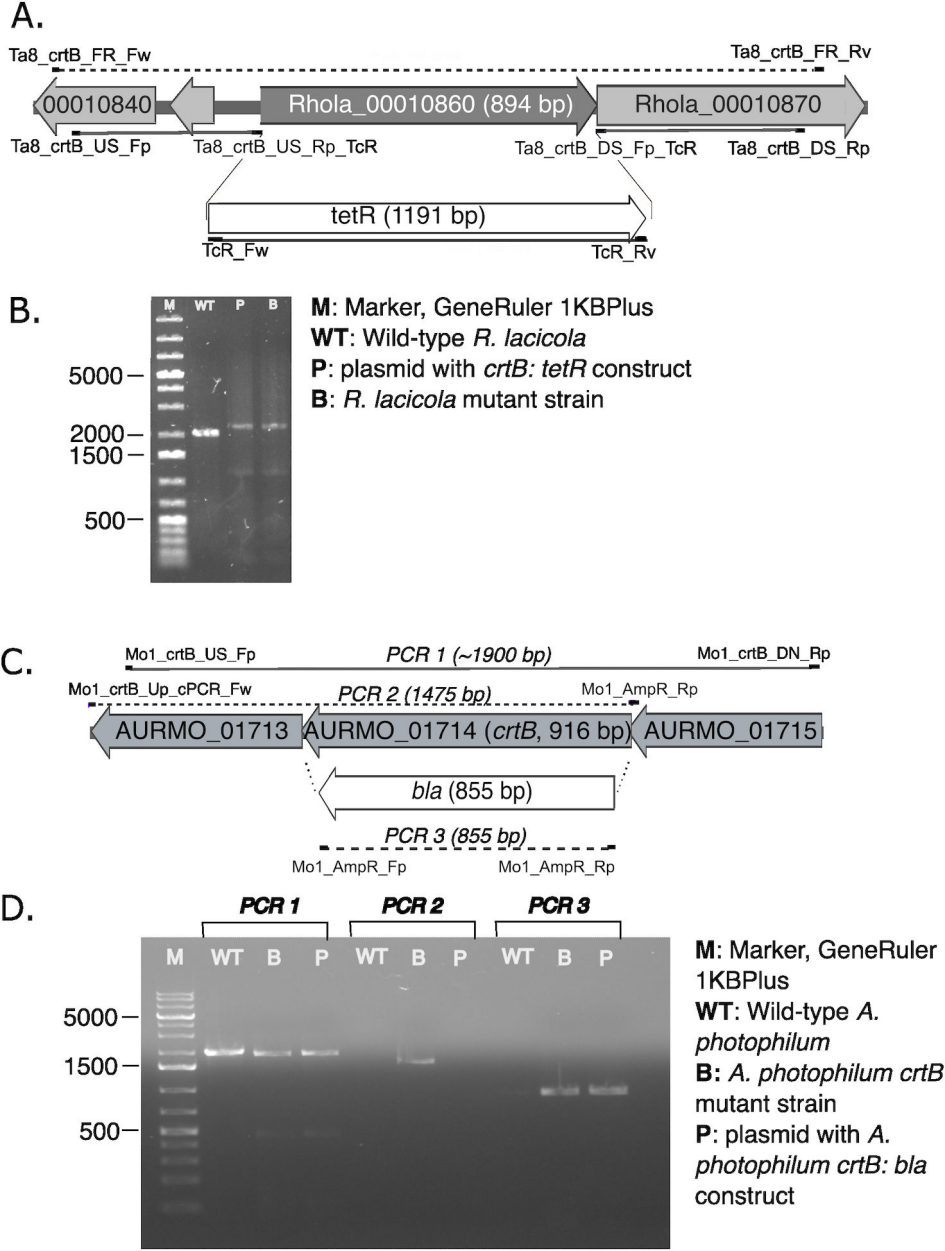
Inactivation of *crtB* by natural transformation and double homologous recombination in *R. lacicola* and *A. photophilum*, replacing *crtB* with *tetR* and *bla*, respectively. Primer sequences can be found in Tables S1 and S2. (A) Inactivation construct for *R. lacicola*. Primers Ta8_crtB_FR_Fw and Ta8_crtB_FR_Rv were used to evaluate mutants (dashed gray line). (B) PCR confirmation of *tetR* insertion into the *crtB* locus. The molecular weight marker used was GeneRuler 1 KB Plus (ThermoFisher, catalog no. SM1331). The size of the amplicon in the putative mutant strain is the same as the amplicon from the plasmid, indicating the replacement of the 894 bp *crtB* gene with the 1,191 bp *tetR* gene. (C) Inactivation construct for *A. photophilum*. Primers Mo1_crtB_Up_cPCR_Fw and Mo1_AmpR_Rp were used to evaluate mutants (dashed gray line). (D) PCR confirmation of *bla* insertion into the *crtB* locus. The size of the *crtB* amplicon in the putative mutant strain is similar in size to the amplicon from the plasmid and wild type, as expected (PCR1). However, amplification using one primer outside of *crtB* and one complementary to *bla* yields a product only in the mutant, indicating replacement of the *crtB* gene in the mutant strain. Amplification with *bla*-specific primers yields identical products from the mutant and plasmid, but no product from wild-type *A. photophilum*.

For a proof of principle experiment, the *crtB* gene encoding phytoene synthase, the first committed step in carotenoid biosynthesis, was targeted for inactivation. A linear construct was made in which the gene encoding tetracycline resistance (*tetR* from pEX18-Tc) was placed in-frame between the regions immediately upstream and downstream of the *crtB* gene from the *R. lacicola* genome (Fig. 1A). *R. lacicola* was transformed using natural transformation, and tetracycline-resistant transformants were white rather than pink in color, demonstrating that pigment biosynthesis was successfully abolished in those strains. PCR on white colonies subsequently confirmed the insertion of the selectable marker in each case (Fig. 1B). No PCR products corresponding to the wild-type genotype were detected, suggesting that the mutation was completely segregated.

The *crtB* gene in *A. photophilum* was inactivated using the same approach, with the *bla* gene inserted between the homology arms upstream and downstream of *crtB* (Fig. 1C). PCR reactions using white colonies as templates again confirmed the insertion of *bla* in the correct location (Fig. 1D).

### Confirmation of loss of *crtB* activity

**Fig 2:**
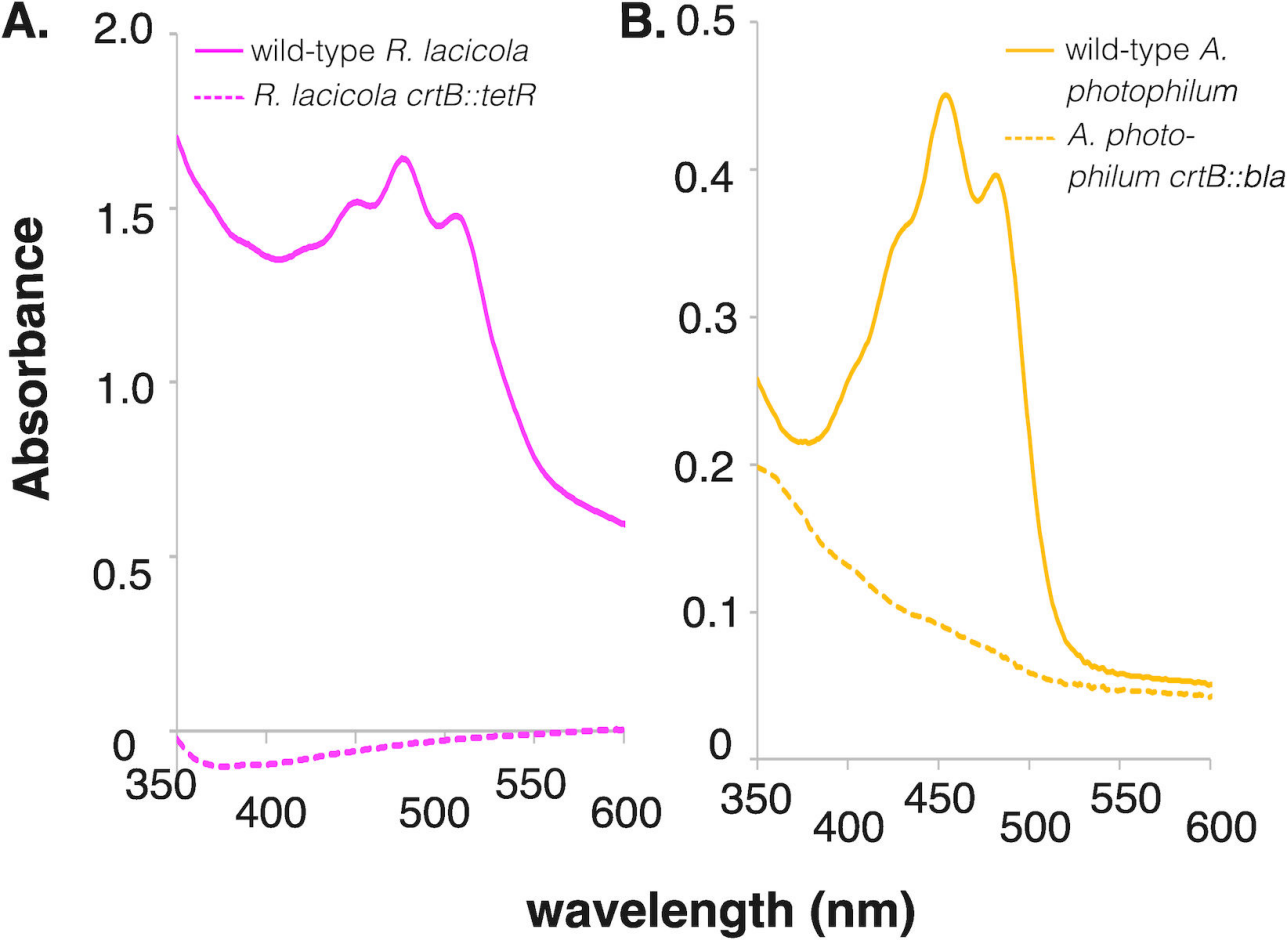
Pigment analysis of *crtB* mutants. Cells were grown to late stationary phase and pigments were extracted in acetone:methanol (7:2 vol/vol), then absorption was measured using a UV/Vis spectrophotometer. (**A**) Wild-type *R. lacicola* makes pink carotenoid pigments (solid line), but the signal from the carotenoids disappears when *crtB* is inactivated (dashed line) since it encodes the first committed step in carotenoid biosynthesis. (**B**) Wild-type *A. photophilum* makes yellow carotenoid pigments (solid lines), which disappear when *crtB* is inactivated (dashed line).

Wild-type *R. lacicola* synthesizes pink carotenoids and wild-type *A. photophilum* synthesizes yellow carotenoids, which have not yet been characterized (19, 20, 38). The first committed step in carotenoid biosynthesis is the synthesis of phytoene from two molecules of geranylgeranyl diphosphate by CrtB, the phytoene synthase (41), so the loss of *crtB* should result in loss of pigmentation. The *crtB* mutants of both strains are colorless. The absorption spectra of the pigments extracted from wild-type cells have maxima between 460 and 530 nm, consistent with mixtures of C40 and C50 carotenoids (Fig. 2). These peaks are absent from the absorption spectra of the Δ*crtB* mutants, indicating that phytoene synthase activity was successfully disrupted.

### Other selectable markers tested in *R. lacicola*

In addition to tetracycline (*tetR* amplified from plasmid pEX18-TC (42)) for antibiotic selection, we tested the kanamycin resistance cassette *npt-ii* from plasmid pEB001 (43), the chloramphenicol resistance gene *cat* from pMCL200 (44), and the beta-lactamase *bla* encoding ampicillin resistance from pUC19 as selectable markers for double homologous recombination. In all four cases, recombinant strains were selected and PCR confirmed that the gene replacement had occurred. However, none of these strains survived either with or without antibiotic selection for more than two rounds of restreaking.

### Electroporation of *R. lacicola*

**Fig 3:**
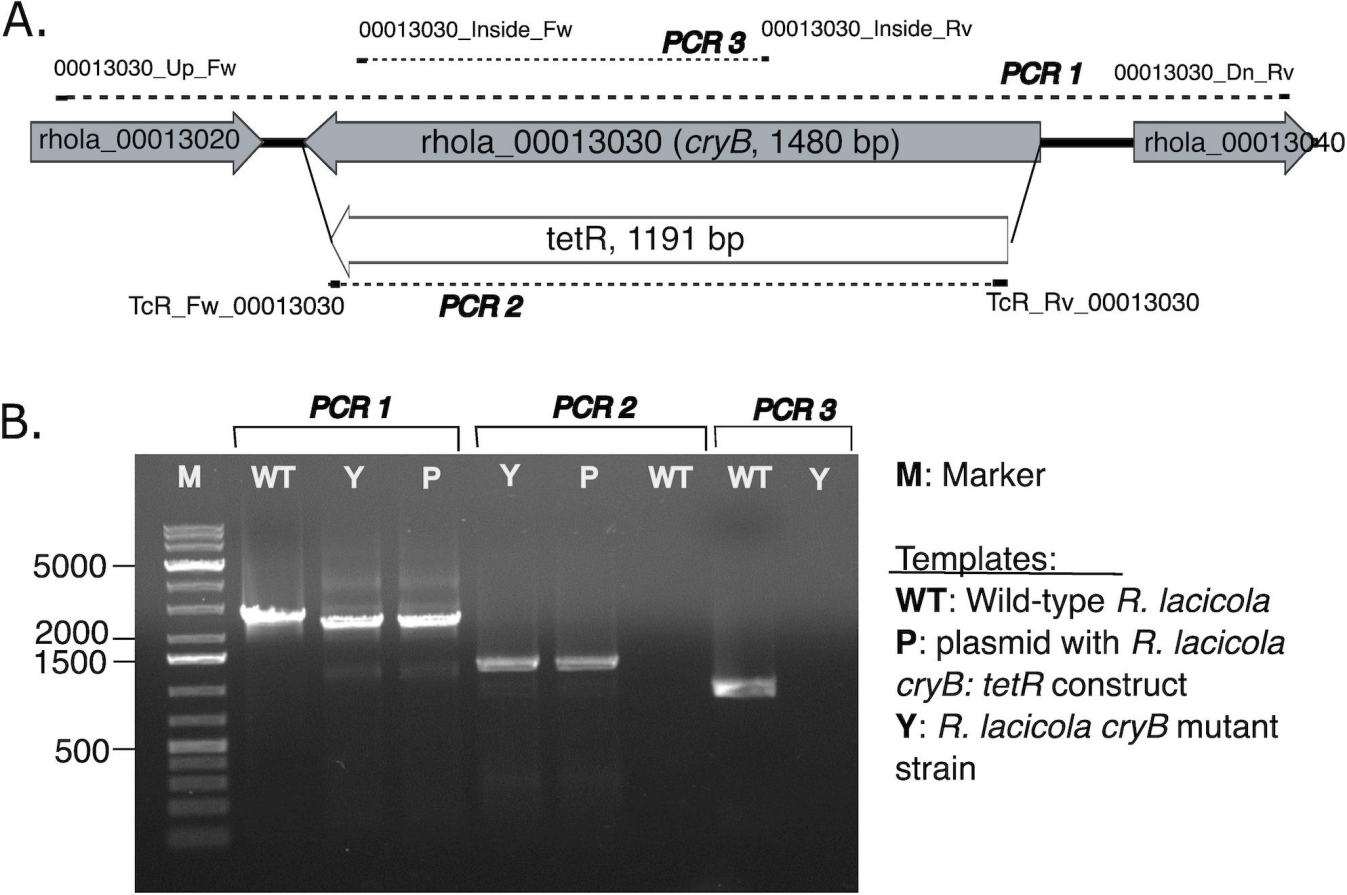
Inactivation of *cryB* by electroporation and double homologous recombination in *R. lacicola*. (A) Construct for replacement of the *cryB* gene, encoding a putative cryptochrome, with the tetracycline resistance gene from plasmid pEX18-Tc. (B) PCR across the insertion site confirms that in the mutant, the product is ~200 bp smaller than in the wild type (PCR1), that the *tetR* gene is present in the mutant but not the wild type (PCR2), and that an internal fragment of *cryB* is present in the wild type but not the mutant strain (PCR3).

To test the efficacy of electroporation as a transformation method, a plasmid construct was made for the inactivation of *rhola_00013030*, encoding a putative cryptochrome potentially relevant to light capture and utilization in *R. lacicola*. Tetracycline-resistant colonies were obtained after one round of selection, and after a subsequent round of growth on non-selective NSY, PCR using a pair of primers complementary to a region of chromosomal DNA outside the construct was used to demonstrate insertion of *tetR* into the chromosome at the appropriate place (Fig. 3).

## DISCUSSION

Actinobacteria are abundant, cosmopolitan, heterotrophic members of freshwater ecosystems, and they regulate their organic carbon metabolism in response to both biotic and abiotic parameters. Extensive (meta) genomic analysis of these bacteria has predicted a variety of genotype-phenotype relationships (18, 24, 28, 40). Here, we demonstrate that the freshwater Actinobacteria *R. lacicola* and *A. photophilum* are naturally transformable and that gene inactivation via homologous recombination is feasible in both strains. We hope that the availability of methods for genome editing in model organisms from two clades within this group will catalyze research that directly tests cause and effect hypotheses between genetic content and phenotype in freshwater ecosystems. Additionally, there is broad interest in exploiting microbes and microbial consortia in freshwater systems to remove contaminants, produce biofuels, or provide ecosystem services (1, 45–47). The tools provided here make it feasible to modify the genomes of some of the most abundant microbes in freshwater communities, a necessary step in engineering synthetic microbial communities in those environments.

Targeted gene inactivation succeeded in *R. lacicola* using selection with a *tetR* (encoding tetracycline resistance) cassette on complex medium amended with tetracycline. Electroporation can be used to transform *R. lacicola*. Additionally, our prior work found that transcripts for a DNA uptake system encoded by *comEC* are more abundant in the dark (40), suggesting that both strains might be naturally transformable. Here, we confirm that if electroporation is not available, both *A. photophilum* and *R. lacicola* can take up DNA during a dark incubation. Both PCR products and plasmid DNA are stable in the cells for enough time that homologous recombination is feasible. Additionally, no special equipment is needed for natural transformation, making these convenient and accessible model organisms.

Direct selection can be used to select for double homologous recombination in both strains. Because this method requires that the selectable markers be expressed from the chromosome, this method also demonstrates that the expression of heterologous genes from the chromosome is feasible. These genome-editing tools can now be used to investigate the roles of specific genes and combinations of genes in freshwater Actinobacterial physiology, both by inactivating genes of interest in *R. lacicola* and *A. photophilum* or by expressing genes of interest from other freshwater Actinobacteria in a phylogenetically related, physiologically relevant host.

We also tested the replacement of *crtB* in *R. lacicola* with genes encoding resistance to ampicillin, chloramphenicol, and kanamycin. Although preliminary data after one round of selection on solid medium with antibiotics indicated successful gene replacement via double homologous recombination, the mutant strains did not survive more than two rounds of restreaking on selective medium. In the case of tetracycline resistance, the mutants are stable on non-selective medium after one round of selection, and the same may be true for the other selectable markers. This may not be a strong enough selection to inactivate genes whose loss would be deleterious to the cell, and might instead select for merodiploidy (48). It also suggests that until a stable plasmid replicon is found for these strains, genes for heterologous expression will have to be inserted into the chromosome. We suggest that the *crtB* locus may be suitable for this, since its inactivation has no apparent effect on growth, and replacement of *crtB* has a visually identifiable phenotype.

Carotenoid pigments appear to be universal in freshwater Actinobacteria and carotenoid biosynthetic pathways have been predicted in several strains based on genome and metagenome-assembled genome sequences (35, 37, 49, 50). However, these pathways have not yet been biochemically characterized, with the exception of two beta-carotene cleavage dioxygenases that produce retinal (35, 37). Here, we demonstrate that in both strains, inactivation of the predicted phytoene synthase, *crtB*, led to the loss of all pigment production. This result confirms that *crtB* indeed encodes a phytoene synthase, and indicates that all of the pigments in both *R. lacicola* and *A. photophilum* are carotenoid pigments. This also confirms our prior result that carotenoid production is not required for viability under ordinary laboratory conditions (38). Inactivation of any gene or combination of genes in this pathway should therefore be feasible, enabling follow-up genetic studies of carotenoid biosynthetic pathways in freshwater species.

Additional follow-up studies investigating fundamental processes, ecological interactions, and potential applications in freshwater Actinobacteria are now possible. Targeted gene inactivation and physiological comparisons to wild type can be used to better understand the role(s) of the actinorhodopsins and heliorhodopsins commonly identified in freshwater Actinobacteria (22, 36, 37, 50, 51) or the identities of the genes required for their unique cell shape (12). The very small genomes of freshwater Actinobacteria appear to change and rearrange rapidly, suggesting a role for horizontal gene transfer (18): now, the importance of the predicted *comEC* competence genes for DNA uptake can be tested. The freshwater Actinobacteria appear to be resistant to grazing (7, 52, 53), so the contributions of specific membrane components to grazing resistance can now be investigated, as can the roles of specific transporters in organic carbon uptake and processing, which may mediate metabolic interactions with other organisms (18).

As members of a ubiquitous and abundant keystone clade, freshwater Actinobacteria likely play important roles in supporting other microbes in highly diverse freshwater environments (54). Targeted gene inactivation or insertion of specific genes into the chromosome for heterologous expression can now be used to investigate the mechanisms of survival, adaptation, and interaction that freshwater Actinobacteria use to thrive in this enormous range of environments, in communities with wide varieties of other microbes. As we use outdoor systems for the synthesis of high-value products (47, 55, 56) or removal of contaminants (57, 58), engineering the microbial communities around the strains with traits of interest may enhance and stabilize the desired activities. Genome-editing tools for more of these “supporting” species could therefore enable better bioproduction in mixed communities.

In sum, here we establish *R. lacicola* and *A. photophilum* as convenient model organisms for investigating the genetics and physiology of freshwater Actinobacteria. Linear and plasmid DNA can be transformed into *R. lacicola* using either natural transformation or electroporation. We have developed selectable markers for each strain, driven by the native *crtB* promoters, for targeted gene inactivation via double homologous recombination. We anticipate that other antibiotic-based selections will soon be usable in this strain, and hope that these tools will be widely used. Although *A. photophilum* is sensitive to fewer antibiotics than *R. lacicola*, it is naturally transformable with linear DNA, and *bla* works as a selectable marker for insertional gene inactivation in this strain. These tools will enable genome engineering in freshwater Actinobacteria for fundamental genetic and ecophysiological characterization as well as for application in engineered freshwater microbial communities.

## SUPPLEMENTAL MATERIAL

Supplemental tables aem.00865-24-s0001.docx Tables S1 and S2.

ASM does not own the copyrights to Supplemental Material that may be linked to, or accessed through, an article. The authors have granted ASM a non-exclusive, world-wide license to publish the Supplemental Material files. Please contact the corresponding author directly for reuse.
