# Supplementary material for: Genome editing in ubiquitous freshwater Actinobacteria
Source: Appl Environ Microbiol. 2024 Oct 16;90(11):e00865-24. doi: 10.1128/aem.00865-24 (PMC11577799; doi:10.1128/aem.00865-24)
Supplement: Supplemental tables — Tables S1 and S2. [file aem.00865-24-s0001.docx]

**Table S1. Primer sequences used for mutant construction and confirmation in *R. lacicola*.** Underlined parts of the primers are complementary to the indicated template.

| **Primer Name** | **Sequence** | **Template** | **Binding location on Template** | **Product and Product size** |
| --- | --- | --- | --- | --- |
| Ta8_crtB_US_Fp | gcctgcaggtcgactctagagggtatccgccagcagcgtgc | *R. lacicola rhola_00010880 (crtB)* | 1,091,248 – 1,091,271 | Region upstream of *crtB* in *R. lacicola*  500 bp |
| Ta8_crtB_US_Rp | ttctggctcacttaggggacggtttctagattacccgcccaaagttgcg | *R. lacicola rhola_00010880 (crtB)* | 1,091,723 – 1,091,754 |  |
| Ta8_crtB_US_Rp_TcR | agcgcattgttagatttcatggtttctagattacccgcccaaagttgcg | *R. lacicola rhola_00010880 (crtB)* | 1,091,723 – 1,091,751 | For confirming insertion in *crtB* of *R. lacicola* |
| Ta8_crtB_DS_Fp_TcR | gccgggccacctcgacctgagtcccctaagtgagccagaaaactgctg | *R. lacicola rhola_00010880 (crtB)* | 1,092,633 – 1,092,660 | Region downstream of *crtB* in *R. lacicola*  500 bp |
| Ta8_crtB_DS_Rv | attcgagctcggtacccgggcgagcgagcctagccagcac | *R. lacicola rhola_00010880 (crtB)* | 1,093,113 – 1,093,132 |  |
| TcR_Fw | gggcgggtaatctagaaaccatgaaatctaacaatgcgctcatcgtcatc | pEX18-Tc *tetR* | 6,046 – 6,075 | Tetracycline resistance gene from plasmid pEX18-TC  1191 bp |
| TcR_Rv | ttctggctcacttaggggactcaggtcgaggtggcccg | pEX18-Tc *tetR* | 4.885 – 4,902 |  |
| Ta8_crtB_FR_Fw | gtgcattccctcgtggccc | *R. lacicola rhola_00010880 (crtB)* | 1,091,190 – 1,091,208 | For confirming deletion of *crtB*  2,036 bp |
| Ta8_crtB_FR_Rv | cactgccggaaaatctagaatcttgcg | *R. lacicola rhola_00010880 (crtB)* | 1,093,200 – 1,093,226 |  |
| 00013030_Up_Fw_TcR | gcctgcaggtcgactctagaggtttgttcaacggcatggatgac | *R. lacicola rhola_00013030 (cryB)* | 1,328,655 – 1,328,678 | Region upstream of *cryB (rhola_00013030)* in *R. lacicola*  497 bp |
| 00013030_Up_Rv_TcR | gccgggccacctcgacctgattccaaaccagtaaatcgcctaggc | *R. lacicola rhola_00013030 (cryB)* | 1,329,130 – 1,329,152 |  |
| TcR_Fw_00013030 | ggcgatttactggtttggaatcaggtcgaggtggccc | pEX18_Tc | 4,885 – 4,901 | Tetracycline resistance gene from plasmid pEX18-TC  1190 bp |
| TcR_Rv_00013030 | agggttagtcggtttagcgcatgaaatctaacaatgcgctcatcgtc | pEX18_Tc | 6,049 – 6,075 |  |
| 00013030_Dn_Fw_TcR | agcgcattgttagatttcatgcgctaaaccgactaacccttg | *R. lacicola rhola_00013030 (cryB)* | 1,330,631 – 1,330,655 | Region downstream of *cryB (rhola_00013030)* in *R. lacicola*  502 bp |
| 00013030_DN_Rv | attcgagctcggtacccggggtgaccaagacctacggtgttc | *R. lacicola rhola_00013030 (cryB)* | 1,331,112 – 1,331,133 |  |
| 00013030_Inside_Fw | cggtgattccttacgaaggtctc | *R. lacicola rhola_00013030 (cryB)* | 1,329,258 – 1,329,280 | For confirming deletion of *cryB (rhola_00013030)*  832 bp |
| 00013030_Inside_Rv | ggccgccgtatctagagc | *R. lacicola rhola_00013030 (cryB)* | 1,330,072 – 1,330,089 |  |

**Table S2. Primer sequences used for mutant construction and confirmation in *A. photophilum.*** Underlined parts of the primers are complementary to the indicated template.

| **Primer Name** | **Sequence** | **Template** | **Binding location on Template** | **Product size** |
| --- | --- | --- | --- | --- |
| Mo1_pEX_Inv_Rp | gttctgcgtcgcacacccaaGACCTGCAGGCATGCAAGCTT | pEX18-Tc | 3,555 – 3,575 | Amplification of pEX18-TC plasmid backbone, for cloning  6,331 bp |
| Mo1_pEX_Inv_Fp | aacaatctcttcgatgctggGGTACCGAGCTCGAATTCGTAATCATGG | pEX18-Tc | 3,594 – 3,621 |  |
| Mo1_crtB_US_Fp | AGCTTGCATGCCTGCAGGTCttgggtgtgcgacgcagaac | *A. photophilum* MWH-Mo1 | 1,680,994 – 1,681,013 | Region upstream of *crtB (AURMO_1714)* in *A. photophilum* strain MWH-Mo1  500 bp |
| Mo1_crtB_US_Rp | CACTGATTAAGCATTGGTAAatgaccaagtccgtagtcattattggtgg | *A. photophilum* MWH-Mo1 | 1,681,465 – 1,681,493 |  |
| Mo1_AmpR_Fp | atgactacggacttggtcatTTACCAATGCTTAATCAGTGAGGCACC | *pUC19* | 1,626 – 1,652 | Beta-lactamase (*bla*) from pUC19  855 bp |
| Mo1_AmpR_Rp | tcgtggagcgcgtgcgatgaATTCAACATTTCCGTGTCGCCCTTATTCC | *pUC19* | 2,452 – 2,480 |  |
| Mo1_crtB_DN_Fp | GCGACACGGAAATGTTGAATtcatcgcacgcgctcca | *A. photophilum* MWH-Mo1 | 1,682,401 – 1,682,417 | Region downstream of *crtB (AURMO_1714)* in *A. photophilum* strain MWH-Mo1  500 bp |
| Mo1_crtB_DN_Rp | ACGAATTCGAGCTCGGTACCccagcatcgaagagattgttcacatgg | *A. photophilum* MWH-Mo1 | 1,682,874 – 1,682,900 |  |
| AmpR-Fp | GCCTGACTCCCCGTCGTGTAGAT | pUC19 | 1,690 – 1,712 | For confirming presence of *bla* in mutant strain  731 bp |
| AmpR-Rp | TCACCAGCGTTTCTGGGTGAGC | pUC19 | 2,399 – 2,420 |  |
| Mo1_crtB_Up_cPCR_Fw | cgtgatgaaggggctagaacc | *A. photophilum* MWH-Mo1 | 1,680,873 – 1,680,893 | For confirming deletion of *crtB (AURMO_1714)* in mutant strain  1375 bp in mutant only; no product in WT |
| Mo1_AmpR_Rp | tcgtggagcgcgtgcgatgaATTCAACATTTCCGTGTCGCCCTTATTCC | pUC19 | 2,452 – 2,480 |  |
